# Supplementary material for: Drug repurposing for aging research using model organisms
Source: Aging Cell. 2017 Jun 16;16(5):1006–15. doi: 10.1111/acel.12626 (PMC5595691; doi:10.1111/acel.12626)
Supplement: Supplementary file 7 — Data S1 Zip‐Archive of all report cards. [file ACEL-16-1006-s007.zip › RC_GD9.pdf]

GD9

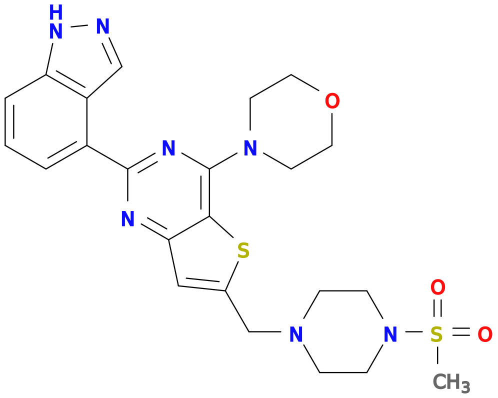

Database identifiers

ChEMBLCompound	CHEMBL521851
CHEBI	65326
ZINC	ZINC16052714
eMolecules	30156208

Ranking

	Rank	Score
Drosophila	98/697	0.791
C. elegans	268/591	0.208

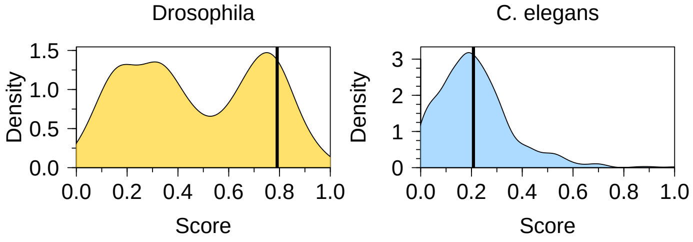

	Ageing implication	Domain conservation	Binding site conservation	Binding affinity	Bioavailability	Lipinski	Promiscuity	Purchasability	Drug approval	**Total**
Drosophila	1.0	0.913	0.957	0.944	(0.9)	-0.05	-0.001	0.1	0.0	0.791
C. elegans	1.0	0.761	0.873	0.944	0.254	-0.05	-0.001	0.1	0.0	0.208

Names

- **pictrelisib**
- Gdc0941
- Pictilisib

Roles

EC 2.7.1.137 (phosphatidylinositol 3-kinase) inhibitor

Status

Approved drug (according to ChEMBL)	No
Number of Rule of 5 violations	1
Binding affinity to original target in log units (RF-Score prediction)	7.82
Burns *C. elegans* bioavailability prediction	-1.91

Compound Target Characteristics

Phosphatidylinositol 4,5-bisphosphate 3-kinase catalytic subunit delta isoform

Best gene implication in ageing for this target family came from gene Q94125 annotated in UniProt release 2014_02. Annotation GO 8340 (determination of adult lifespan) was Inferred from Mutant Phenotype

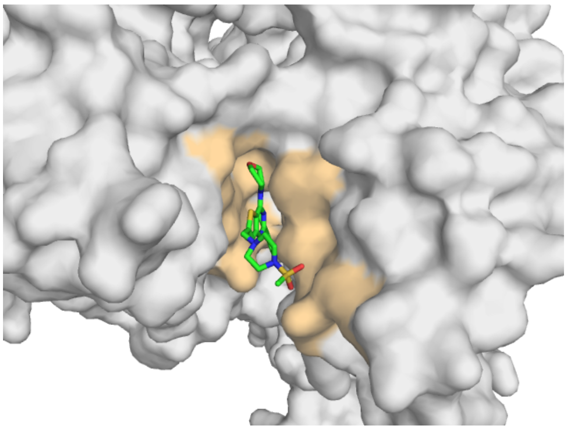

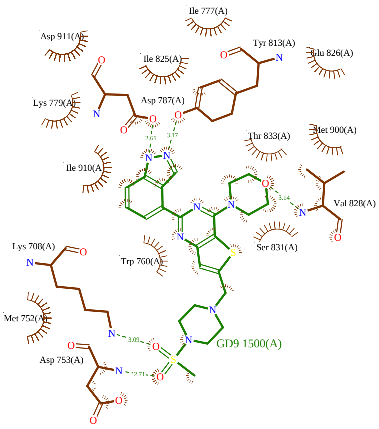

protein	amino acids contacts (binding site)															
PDB:2wxp:chainA:O35904	K	F	M	D	W	I	K	D	Y	I	E	V	S	T	M	I	D
tr:B7ZM44:B7ZM44_HUMAN	K	F	M	D	W	I	K	D	Y	I	E	V	S	T	M	I	D
sp:O00329:PK3CD_HUMAN	K	F	M	D	W	I	K	D	Y	I	E	V	S	T	M	I	D
tr:Q5SR50:Q5SR50_HUMAN	K	F	M	D	W	I	K	D	Y	I	E	V	S	T	M	I	D
tr:D4A5Q1:D4A5Q1_RAT	K	F	M	D	W	I	K	D	Y	I	E	V	S	T	M	I	D
tr:Q3TBW3:Q3TBW3_MOUSE	K	F	M	D	W	I	K	D	Y	I	E	V	S	T	M	I	D
tr:Q3T9Y0:Q3T9Y0_MOUSE	K	F	M	D	W	I	K	D	Y	I	E	V	S	T	M	I	D
sp:O35904:PK3CD_MOUSE	K	F	M	D	W	I	K	D	Y	I	E	V	S	T	M	I	D
tr:Q3UDT3:Q3UDT3_MOUSE	K	F	M	D	W	I	K	D	Y	I	E	V	S	T	M	I	D
tr:Q8CI98:Q8CI98_MOUSE	K	F	M	D	W	I	K	D	Y	I	E	V	S	T	M	I	D
tr:B0QZL5:B0QZL5_MOUSE	K	F	M	D	W	I	K	D	Y	I	E	V	S	T	M	I	D
tr:P91634:P91634_DROME	K	V	M	D	W	I	K	D	Y	I	E	V	A	T	M	I	D
tr:H1ZY88:H1ZY88_DROME	K	V	M	D	W	I	K	D	Y	I	E	V	A	T	M	I	D
tr:H1ZY91:H1ZY91_DROME	K	V	M	D	W	I	K	D	Y	I	E	V	A	T	M	I	D
sp:Q94125:AGE1_CAEEL	T	V	L	G	M	I	K	D	Y	I	E	V	C	T	M	I	D

	whole protein		domain-based		contact-based	
protein	ident	simil	ident	simil	ident	simil
PDB:2wxp:chainA:O35904	1.0	1.0	1.0	1.0	1.0	1.0
tr:B7ZM44:B7ZM44_HUMAN	0.95	0.98	0.97	0.99	1.0	1.0
sp:O00329:PK3CD_HUMAN	0.95	0.98	0.97	0.99	1.0	1.0
tr:Q5SR50:Q5SR50_HUMAN	0.86	0.9	0.97	0.99	1.0	1.0
tr:D4A5Q1:D4A5Q1_RAT	0.89	0.9	0.9	0.91	1.0	1.0
tr:Q3TBW3:Q3TBW3_MOUSE	1.0	1.0	1.0	1.0	1.0	1.0
tr:Q3T9Y0:Q3T9Y0_MOUSE	1.0	1.0	1.0	1.0	1.0	1.0
sp:O35904:PK3CD_MOUSE	1.0	1.0	1.0	1.0	1.0	1.0
tr:Q3UDT3:Q3UDT3_MOUSE	1.0	1.0	1.0	1.0	1.0	1.0
tr:Q8CI98:Q8CI98_MOUSE	1.0	1.0	1.0	1.0	1.0	1.0
tr:B0QZL5:B0QZL5_MOUSE	1.0	1.0	1.0	1.0	1.0	1.0
tr:P91634:P91634_DROME	0.37	0.75	0.52	0.84	0.88	0.91
tr:H1ZY88:H1ZY88_DROME	0.37	0.75	0.52	0.84	0.88	0.91
tr:H1ZY91:H1ZY91_DROME	0.37	0.75	0.52	0.83	0.88	0.91
sp:Q94125:AGE1_CAEEL	0.23	0.62	0.34	0.72	0.65	0.76

Pi3K92E (FBgn0015279) associated phenotypes

RU486 conditional, cell autonomous, cell death defective, cell growth defective, cell non-autonomous, cell size defective, chemical resistant, chemical sensitive, decreased cell death, decreased cell growth, decreased cell number, decreased cell size, developmental rate defective, feeding behavior defective,

heat sensitive, hyperplasia, increased cell growth, increased cell number, increased cell size, large body, locomotor behavior defective, long lived, neuroanatomy defective, neurophysiology defective, nutrition conditional, partially lethal - majority die, photoperiod response variant, planar polarity defective, short lived, size defective, small body, smell perception defective, somatic clone, some die during pupal stage, starvation stress response defective, stress response defective, wound healing defective

(Information from FlyBase)

age-1 (WBGene00000090) associated phenotypes

DMPP resistant, aging variant, brood size variant, cadmium resistant, dauer arrest variant, dauer constitutive, egg laying variant, extended life span, failure to hatch, fat content increased, hermaphrodite fertility variant, larval lethal, late stage egg laid, life span variant, organism physiology variant, organism temperature response variant, paraquat resistant, reproductive longevity extended, scrawny, sterile, transgene subcellular localization variant

(Information from WormBase)

age-1 (UniProt:Q94125) annotation

Function: Phosphatidylinositol 3-kinase homolog that regulates longevity and diapause. Could function in the development or neuroendocrine signaling of the dauer pathway (PubMed:8700226). Mediates susceptibility to enteropathogenic E.coli infection (PubMed:16091039). (PubMed:16091039, PubMed:8700226).

Developmental stage: Expressed both maternally and zygotically. (PubMed:8700226).

(Information from UniProt)

Phosphatidylinositol 4,5-bisphosphate 3-kinase catalytic subunit beta isoform

Best gene implication in ageing for this target family came from gene Q94125 annotated in UniProt release 2014_02. Annotation GO 8340 (determination of adult lifespan) was Inferred from Mutant Phenotype

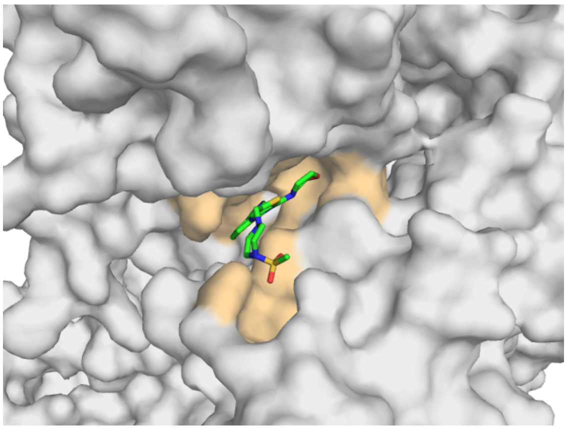

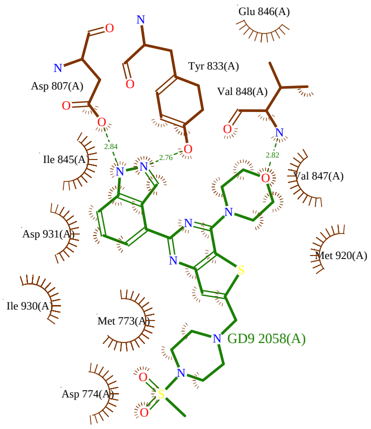

protein	amino acids contacts (binding site)
PDB:2y3a:chainA:Q8BTI9	Y M D D Y I E V V M I D
tr:Q68DL0:Q68DL0_HUMAN	Y M D D Y I E V V M I D
tr:B4DZI3:B4DZI3_HUMAN	Y M D D Y I E V V M I D
sp:P42338:PK3CB_HUMAN	Y M D D Y I E V V M I D
tr:G3V839:G3V839_RAT	Y M D D Y I E V V M I D
tr:Q9CTK7:Q9CTK7_MOUSE	Y M D D Y I E V V M I D
tr:Q3TNJ8:Q3TNJ8_MOUSE	Y M D D Y I E V V M I D
tr:A0JNZ1:A0JNZ1_MOUSE	Y M D D Y I E V V M I D
sp:Q8BTI9:PK3CB_MOUSE	Y M D D Y I E V V M I D
tr:P91634:P91634_DROME	V M D D Y I E V V M I D
tr:H1ZY88:H1ZY88_DROME	V M D D Y I E V V M I D
tr:H1ZY91:H1ZY91_DROME	V M D D Y I E V V M I D
sp:Q94125:AGE1_CAEEL	V L G D Y I E V V M I D

	whole protein		domain-based		contact-based	
protein	ident	simil	ident	simil	ident	simil
PDB:2y3a:chainA:Q8BTI9	1.0	1.0	1.0	1.0	1.0	1.0
tr:Q68DL0:Q68DL0_HUMAN	0.47	0.48	0.97	0.98	1.0	1.0
tr:B4DZI3:B4DZI3_HUMAN	0.6	0.61	0.98	1.0	1.0	1.0
sp:P42338:PK3CB_HUMAN	0.95	0.98	0.98	1.0	1.0	1.0
tr:G3V839:G3V839_RAT	0.98	0.99	0.99	1.0	1.0	1.0
tr:Q9CTK7:Q9CTK7_MOUSE	0.3	0.3	0.87	0.87	1.0	1.0
tr:Q3TNJ8:Q3TNJ8_MOUSE	0.31	0.31	0.92	0.92	1.0	1.0
tr:A0JNZ1:A0JNZ1_MOUSE	0.98	0.98	1.0	1.0	1.0	1.0
sp:Q8BTI9:PK3CB_MOUSE	1.0	1.0	1.0	1.0	1.0	1.0
tr:P91634:P91634_DROME	0.36	0.74	0.52	0.83	0.92	0.96
tr:H1ZY88:H1ZY88_DROME	0.36	0.74	0.52	0.83	0.92	0.96
tr:H1ZY91:H1ZY91_DROME	0.36	0.74	0.52	0.83	0.92	0.96
sp:Q94125:AGE1_CAEEL	0.22	0.62	0.34	0.71	0.75	0.87

Pi3K92E (FBgn0015279) associated phenotypes

RU486 conditional, cell autonomous, cell death defective, cell growth defective, cell non-autonomous, cell size defective, chemical resistant, chemical sensitive, decreased cell death, decreased cell growth, decreased cell number, decreased cell size, developmental rate defective, feeding behavior defective, heat sensitive, hyperplasia, increased cell growth, increased cell number, increased cell size, large body, locomotor behavior defective, long lived, neuroanatomy defective, neurophysiology defective, nutrition conditional, partially lethal - majority die, photoperiod response variant, planar polarity defective, short lived, size defective, small body, smell perception defective, somatic clone, some die during pupal stage, starvation stress response defective, stress response defective, wound healing defective

(Information from FlyBase)

age-1 (WBGene00000090) associated phenotypes

DMPP resistant, aging variant, brood size variant, cadmium resistant, dauer arrest variant, dauer constitutive, egg laying variant, extended life span, failure to hatch, fat content increased, hermaphrodite fertility variant, larval lethal, late stage egg laid, life span variant, organism physiology variant, organism temperature response variant, paraquat resistant, reproductive longevity extended, scrawny, sterile, transgene subcellular localization variant

(Information from WormBase)

age-1 (UniProt:Q94125) annotation

Function: Phosphatidylinositol 3-kinase homolog that regulates longevity and diapause. Could function in the development or neuroendocrine signaling of the dauer pathway (PubMed:8700226). Mediates susceptibility to enteropathogenic E.coli infection (PubMed:16091039). (PubMed:16091039, PubMed:8700226).
Developmental stage: Expressed both maternally and zygotically. (PubMed:8700226).

(Information from UniProt)
